# Elevated Bathing-Associated Disease Risks Despite Certified Water Quality: A Cohort Study

**DOI:** 10.3390/ijerph9051548

**Published:** 2012-04-25

**Authors:** Panagiotis Papastergiou, Varvara Mouchtouri, Ourania Pinaka, Anna Katsiaflaka, George Rachiotis, Christos Hadjichristodoulou

**Affiliations:** Department of Hygiene and Epidemiology, Faculty of Medicine, University of Thessaly, 22 Papakyriazi Str., Larissa 41222, Greece; Email: panpapast@med.uth.gr (P.P.); mouchtourib@med.uth.gr (V.M.); rpinaka@gmail.com (O.P.); akatsaf@med.uth.gr (A.K.); gsrachmed@yahoo.com (G.R.)

**Keywords:** bacterial indicators, bathers, health effects, recreational water, swimming, sea-water quality

## Abstract

Bacteriological water quality criteria have been recommended to ensure bathers’ health. However, this risk-assessment approach is based mainly on routine measurements of fecal pollution indicator bacteria in seawater, and may not be adequate to protect bathers effectively. The aim of this study was to assess the risks of symptoms related to infectious diseases among bathers after exposure to seawater which was of excellent quality according to EU guidelines. This study is a cohort study recruiting bathers and non-bathers. Water samples were collected for estimating bacterial indicators. Univariable and multivariable analysis was performed to compare the risks of developing symptoms/diseases between bathers and non-bathers. A total of 3805 bathers and 572 non-bathers were included in the study. Water analysis results demonstrated excellent quality of bathing water. Significantly increased risks of symptoms related to gastrointestinal infections (OR = 3.60, 95% CI 1.28–10.13), respiratory infections (OR = 1.92, 95% CI 1.00–3.67), eye infections (OR = 2.43, 95% CI 1.27–4.63) and ear infections (OR = 17.21, 95% CI 2.42–122.34) were observed among bathers compared with non-bathers. Increased rates of medical consultation and medication use were also observed among bathers. There was evidence that bathers experienced increased morbidity compared with non-bathers though the bathing waters met bacteriological water quality criteria. These results suggest that risk assessments of recreational seawaters should not only focus on bacteriological water quality criteria.

## Abbreviations

EU: European Union
EPA: US Environmental Protection Agency
OR: odds ratio
RR: relative risk

## 1. Introduction

Epidemiological studies have been carried out since the late 1940s and early 1950s in attempts to define the levels of risk following exposure to different concentrations of bacteria in bathing waters [1]. Most epidemiological studies have been prospective cohort studies, and only a few retrospective cohort studies or randomized controlled trials have been performed [2,3,4,5,6,7,8]. Previous epidemiological studies have investigated the exposure-response relationship between health outcomes and bathing-water quality based on measures of faecal pollution indicator bacteria. The existence of threshold values of indicator-bacteria counts for health outcomes was studied, as well as possible variations in the severities of outcomes associated with microbiological water quality. Bacteriological water quality criteria (fecal pollution bacteria thresholds) have been recommended to ensure bathers’ health. These criteria are based on estimates of bacterial indicator counts and gastrointestinal illness rates that are currently believed to provide a high level of protection for bathers’ health [9,10]. However, it should be pointed out that there is no evidence of “no risk” below advisory levels for swimmers/bathers.The 1976 EU Bathing Water Directive adopted by Greece with the Ministerial Decision 46399/1352/86 included more stringent quality requirements than those in the 1976 EU Directive [11,12]. The new EU Bathing Water Directive of 2006 was adopted in 2009 with the Ministerial Decision 8600/416/E103/2009 [10,13]. The Hellenic Ministry of Environment, Energy and Climate Change (former Ministry for the Environment, Physical Planning and Public Works) is the main competent authority for the implementation of this Directive. Greece is located in the North-Eastern part of the Mediterranean. It includes over 2500 islands and has always been considered to be closely related to the sea. Every year, Greece and its coastal bathing areas welcome millions of foreign tourists, in addition to its own population, and beach monitoring is thus a high priority. Measurements of bacterial indicator counts in Greek coastal waters have largely been found to be in accordance with the values in the guidelines set by the European Directive. In a 10-year analysis of seawater microbiological quality data (231,205 water samples), measurements of *Escherichia coli* (99.6%) and enterococci (100%) were deemed acceptable by EU bathing standards. [14]. However a risk-assessment approach based mainly on routine measurements of fecal pollution indicator bacteria in seawater may not be adequate to protect bathers’ health effectively; human health while bathing in recreational water will ultimately depend on the impact of a combination of physical, chemical and biological hazards [15]. This study formed part of the Greek bathers’ cohort study. The objective was to examine the health effects and risks of infectious diseases among bathers who where bathing in water that met the most strict water quality criteria for bacteria proposed by EU Directives and the US Environmental Protection Agency (EPA) [9,10,11]. 

## 2. Results

### 2.1. Demographics

A total of 4293 bathers (2923 households) were asked to participate in the study. Only 34 bathers (0.79%) refused to participate in the first part of the interview survey. An additional 290 bathers (6.76%) failed to respond to, or refused to take part in the follow-up telephone interview 10 days later (total response rate = 92.45%). Based on our definition of bathers (body emersion for at least 10 min) 3,805 of 3,969 (95.9%) participants were defined as bathers. Among the bathers, 1837 (48.3%) were male and 1947 (51.2%) were female, while for 21 (0.5%) bathers information was not recorded and the mean bathing duration was estimated to be 50 minutes. Among the non-bathers, 243 (42.5%) were male and 329 (57.5%) were female. The mean age of the bathers was 28.33 years old (28.93 in males and 27.84 in females, ranged from 6 months to 98 years) and only five bathers were above 79 years old. The mean age of non-bathers was 39.05 years (41.59 in males and 37.17 in females, ranged from 1 year to 95 years). The majorities of both bathers and non-bathers were resident in the wider area of Thessaly (85.7% and 94.6%, respectively). Selected characteristics of the study population are shown in Table 1.

**Table 1:** Characteristics of bathers and non-bathers.

	Bathers ( *n* = 3805)		Non-bathers ( *n* = 572)		
	No.	(%)	No.	(%)	
**Age (years)**					
0–4	102	(2.7)	19	(3.3)	
5–9	147	(3.9)	23	(4.0)	
10–14	185	(4.9)	26	(4.5)	
15–19	599	(15.7)	38	(6.6)	
20–24	742	(19.5)	69	(12.1)	
25–29	541	(14.2)	33	(5.8)	
30–34	350	(9.2)	34	(5.9)	
35–39	295	(7.8)	54	(9.4)	
40–44	273	(7.2)	50		(8.7)
45–49	210	(5.5)	52		(9.1)
50–54	129	(3.4)	41		(7.2)
55–59	90	(2.4)	33		(5.8)
60–64	39	(1.0)	19		(3.3)
65–69	31	(0.8)	20		(3.5)
70+	46	(1.2)	61		(10.7)
missing	26	(0.6)	0		(0.0)
**Gender**					
Male	1837	(48.3)	243		42.5
Female	1947	(51.2)	329		57.5
missing	21	(0.5)	0		(0.0)
**Residence**					
Larissa area(Locals)	2521	(66.2)	450		(78.7)
Thessaly (except of Larissa area) (Visitors)	726	(19.1)	91		(15.9)
Greece (except of Thessaly area) (Visitors)	543	(14.3)	31		(5.4)
missing	15	(0.4)	0		(0.0)
**Incidence of illness among locals **					
Gastroenteritis (A) ^a^	61	(2.4)	3		(0.7)
Gastroenteritis (B) ^b^	39	(1.6)	2		(0.4)
Respiratory infection (A) ^a^	149	(5.9)	11		(2.4)
Respiratory infection (B) ^b^	52	(2.1)	6		(1.3)
Ear infection (A) ^a^	77	(3.1)	1		(0.2)
Ear infection (B) ^b^	18	(0.7)	0		(0.0)
Eye infection (A) ^a^	132	(5.2)	8		(1.8)
Eye infection (B) ^b^	50	(2.1)	3		(0.7)
Cutaneous infection	12	(0.5)	1		(0.2)
Medical consultation	55	(2.2)	2		(0.4)
Medication receiving	87	(3.5)	5		(1.1)
Hospitalization/home care	4	(0.2)	1		(0.2)
**Incidence of illness among visitors**					
Gastroenteritis (A) ^a^	53	(4.2)	1		(0.8)
Gastroenteritis (B) ^b^	35	(2.8)	1		(0.8)
Respiratory infection (A) ^a^	87	(6.9)	4		(3.4)
Respiratory infection (B) ^b^	31	(2.6)	1		(0.8)
Ear infection (A) ^a^	52	(4.1)	0		(0.0)
Ear infection (B) ^b^	9	(0.7)	0		(0.0)
Eye infection (A) ^a^	73	(5.8)	3		(2.5)
Eye infection (B) ^b^	26	(2.1)	0		(0.0)
Cutaneous infection	9	(0.7)	0		(0.0)
Medical consultation	40	(3.2)	2		(1.6)
Medication receiving	52	(4.1)	1		(0.8)
Hospitalization/home care	1	(0.1)	0		(0.0)

^a^ illness definition A; ^b^ illness definition B.

### 2.2. Microbiological Water Quality

A total of 149 seawater samples were taken and analyzed during the survey on the same day that the interviews took place (67 from beach A, 61 from beach B and 21 from beach C). All microbiological test results conformed to the EU excellent requirements based on a 95th Percentile evaluation as described in Directive 2006/7/EC and the geometric mean as described by the EPA (Table 2). The number of days that the Enterococci exceeded single day maximums was one day. The bathers participated in the study this specific day did not have increased incidence of the symptoms.

**Table 2:** Sea water microbiological analysis results for the three beaches.

Beach	(cfu/100 mL)	ECOL *	FCOL *	TCOL *	*Enterococci*
A	95-Percentile	**14.9**	33.1	58.8	**64.6**
	Geometric mean (range)	**2.2**	2.9	4.5	**5.6 (0–1380)**
	N	67	67	67	67
B	95-percintile	**10.8**	11.2	26.5	**16.3**
	Geometric mean (range)	**1.9**	2.0	3.1	**2.8 (0–74)**
	N	61	61	61	61
C	95-percintile	**4.7**	5.4	17.4	**10.6**
	Geometric mean (range)	**1.6**	1.7	3.0	**2.5 (0–15)**
	N	21	21	21	21

* ECOL = *E. Coli*; FCOL = faecal coliforms; TCOL = total coliforms.

### 2.3. Symptoms Related to Infectious Diseases

The incidences of symptoms possibly related to infectious diseases among bathers and non-bathers are shown in Table 1. The results of univariable and multivariable analyses of all health effects in bathers and non-bathers are presented in Table 3, Table 4, respectively.

**Table 3:** Association of symptoms and related infections among bathers and non-bathers (univariable analysis).

Symptom	Bathers with symptom/total (%)	Non bathers with symptom/total (%)	Relative Risk	*p*-value
Nausea or vomiting	40/3796 (1.1)	0/571 (0.0)	12.20 ^a^	**0.022**
Abdominal pain	74/3796 (1.9)	3/571 (0.5)	3.71	**0.016**
Diarrhea more than two times	39/3796 (1.0)	4/571 (0.7)	1.47	0.461
Fever	11/3796 (0.3)	1/571 (0.2)	1.66	0.626
Gastroenteritis (A)	114/3796 (3.0)	4/571 (0.7)	4.29	**0.002**
Gastroenteritis (B)	74/3778 (2.0)	3/570 (0.5)	3.72	**0.016**
Sore throat	100/3796 (2.6)	3/571 (0.5)	5.01	**0.002**
Dysphagia (odynophagia)	41/3796 (1.1)	2/571 (0.4)	3.08	0.101
Rheum (runny nose)	72/3796 (1.9)	7/571 (1.2)	1.55	0.433
Cough	84/3796 (2.2)	8/571 (1.4)	1.58	0.330
Hoarseness	79/3796 (2.1)	10/571 (1.8)	1.19	0.673
Respiratory infection (A)	236/3796 (6.2)	15/571 (2.6)	2.37	**0.005**
Respiratory illness (B)	83/3643 (2.3)	7/563 (1.2)	1.83	0.236
Ear pain	93/3796 (2.4)	1/571 (0.2)	13.99	**<0.001**
Fullness in the ear	35/3796 (0.9)	0/571 (0.0)	10.70 ^a^	**0.035**
Otorrhea	21/3796 (0.6)	0/571 (0.0)	6.48 ^a^	0.100
Ear itching	16/3796 (0.4)	0/571 (0.0)	4.97 ^a^	0.174
Ear infection (A)	129/3796 (3.4)	1/571 (0.2)	19.40	**<0.001**
Ear infection (B)	27/3694 (0.7)	0/570 (0.0)	8.50 ^a^	0.067
Eye redness	145/3796 (3.8)	3/571 (0.5)	7.27	**<0.001**
Eye pain or burn	91/3796 (2.4)	4/571 (0.7)	3.42	**0.010**
Tear secretion (eyes discharge)	43/3796 (1.0)	7/571 (1.2)	0.92	0.861
Mucopurulent exudates (eye)	25/3796 (0.7)	0/571 (0.0)	7.68 ^a^	0.081
Eye infection (A)	205/3796 (5.4)	11/571 (1.9)	2.80	**0.001**
Eye infection (B)	76/3667 (2.1)	3/563 (0.5)	3.89	**0.012**
Cutaneous infection	21/3796 (0.6)	1/571 (0.2)	3.16	0.233
Urinary tract infection ^b^	6/1944 (0.3)	0/328 (0.0)	2.20 ^a^	0.320
Vaginitis ^b^	24/1944 (1.2)	1/328 (0.3)	4.05	0.135
Medical consultation	94/3796 (2.5)	4/571 (0.7)	3.54	**0.008**
Medication receiving	139/3796 (3.7)	6/571 (1.1)	3.49	**0.001**
Hospitalization/home care	5/3796 (0.1)	1/571 (0.2)	0.75	0.794

^a^ Relative risk (RR) by using Haldane correction; ^b^ only females were included.

**Table 4:** Association of symptoms and related infections among bathers and non-bathers (multivariable analysis).

Symptom	Odds Ratio	95% CI	p-value	AUC ^b^
Abdominal pain	3.16	0.95–10.51	0.061	0.638
Diarrhea more than two times	1.06	0.35–3.21	0.911	0.658
Gastroenteritis (A)	**3.60 ^a^**	**1.28–10.13**	**0.015**	0.632
Gastroenteritis (B)	3.16	0.95–10.52	0.060	0.639
Sore throat	**4.28 ^a^**	**1.35–13.51**	**0.013**	0.653
Dysphagia (odynophagia)	2.45	0.60–10.02	0.213	0.643
Rheum (runny nose)	1.17	0.38–3.66	0.783	0.647
Cough	1.27	0.49–3.26	0.627	0.643
Hoarseness	0.93	0.41–2.14	0.872	0.618
Respiratory infection (A)	**1.92 ^a^**	**1.00–3.67**	**0.049**	0.648
Respiratory infection (B)	1.50	0.53–4.21	0.444	0.672
Ear pain	**12.02 ^a^**	**1.69–85.58**	**0.013**	0.685
Ear infection (A)	**17.21 ^a^**	**2.42–122.34**	**0.004**	0.659
Eye redness	**6.15 ^a^**	**2.00–18.91**	**0.002**	0.651
Eye pain or burn	**3.10 ^a^**	**1.19–8.06**	**0.021**	0.671
Tear secretion	0.82	0.35–1.91	0.641	0.577
Eye infection (A)	**2.43 ^a^**	**1.27–4.63**	**0.007**	0.631
Eye infection (B)	**3.48 ^a^**	**1.16–10.42**	**0.026**	0.654
Cutaneous infection	2.92	0.38–22.62	0.306	0.635
Medical consultation	**3.07 ^a^**	**1.15–8.23**	**0.026**	0.632
Medication receiving	**2.98 ^a^**	**1.30–6.82**	**0.010**	0.615

^a^ Statistically significant; Controlling for age, gender and residence, and clustering within households; ^b^ AUC: Area Under ROC Curve provided by ROC analysis.

### 2.4. Symptoms Related to GI and RI

Both univariable and multivariable analysis identified an increased risk of possible GI among bathers compared to non-bathers according to definition A (OR = 3.60, 95% CI 1.28–10.13), and some indication of increased risk according to definition B (OR = 3.16, 95% CI 0.95–10.52). A significant difference in possible GI was found between local and visiting bathers (OR = 0.56, 95% CI 0.38–0.82).

Also, both univariable and multivariable analysis identified some evidence of an increased risk of sore throat for bathers (OR = 4.28, 95% CI 1.35–13.51). Furthermore, the risk of possible RI was only increased in bathers compared with non-bathers using definition A (OR = 1.92, 95% CI 1.00–3.67), but not using the more specific definition B (Table 3, Table 4).

Moreover, further analysis (logistic regression) among bathers was performed in order to explore the association between selected factors (food consumption, swimming time, head immersion, enterococci density and bather density) and symptoms possibly related to infections after adjustment for gender, age, and residence also taking into account the clustering effect of household. 

The results have shown that food consumption was not associated with an elevated risk of symptoms possibly related to GI infections. Interestingly, swimming time was significantly associated with symptoms possibly related to GI and respiratory infections. In particular, bathers with a swimming time more than 60 min have recorded a 96% increased risk (OR = 1.96, 95% CI 1.17–3.30) of reporting abdominal pain, and a 94% increased risk (OR = 1.94, 95% CI 1.15–3.27) of reporting gastroenteritis according to case definition B. In addition, increased swimming time was associated with an elevated odds ratio for Sore throat (OR = 1.69, 95% CI 1.02–2.82). Finally, swimming time more than 60 min was significantly associated with the report of symptoms related to eye infection (Table 5).

### 2.5. Symptoms Related to Ear Eye and Cutaneous Infections

Univariable and multivariable analysis identified some indication of increased risk of ear pain in bathers (OR = 12.02, 95% CI 1.69–85.58). Some evidence of increased risk of ear infection was also found among bathers compared with non-bathers but only using definition A (OR = 17.21, 95% CI 2.42–122.34) and not the more specific definition B (Table 3, Table 4). 

Bathers were also at increased risk of developing eye redness (OR = 6.15, 95% CI 2.00–18.91) and eye pain or eye burning symptoms (OR = 3.10, 95% CI 1.19–8.06). Univariable and logistic regression analysis also demonstrated an increased risk of possible eye infections among bathers compared to non-bathers using both definitions A (OR = 2.43, 95% CI 1.27–4.63) and B (OR=3.48, 95% CI 1.16–10.42) (Table 3, Table 4). There was an increased relative risk of possible eye infection among bathers who reported immersing their heads in the water (RR = 1.79, 95% CI 1.11–2.88) compared with bathers who did not immerse their heads. 

There was no significant difference in the risk of acquiring cutaneous infections among bathers and non-bathers (OR = 2.92, 95% CI 0.38–22.62).

**Table 5:** Association of symptoms and related exposures only for bathers (multivariable analysis).

	Food consumption			Swimming time ^a^			Head immersion			Enterococci density ^b^			Bather density ^c^		
Symptom	OR	95% CI	*p*	OR	95% CI	*p*	OR	95% CI	*p*	OR	95% CI	*p*	OR	95% CI	*p*
Abdominal pain	1.11	(0.67–1.83)	0.689	**1.96**	(1.17–3.30)	**0.011**	1.35	(0,64–2,86)	0.434	0.53	(0.13–2.23)	0.385	**2.01**	(1.00–4.03)	**0.049**
Diarrhea more than 2 times	0.95	(0.51–1.79)	0.881	1.74	(0.84–3.59)	0.136	0.98	(037–259)	0.960	NA	NA	NA	**4.10**	(1.42–11.80)	**0.009**
Gastroenteritis (A)	1.01	(0.67–1.53)	0.968	1.22	(0.75–1.99)	0.424	0.99	(0.57–1.71)	0.968	0.68	(0.27–1.76)	0.428	1.34	(0.81–2.21)	0.250
Gastroenteritis (B)	1.10	(0.67–1.82)	0.704	**1.94**	(1.15–3.27)	**0.013**	1.35	(0.64–2.86)	0.433	0.53	(0.12–2.22)	0.382	**2.02**	(1.01–4.06)	**0.048**
Sore throat	**0.57**	(0.36–0.89)	**0.013**	**1.69**	(1.02–2.82)	**0.043**	0.89	(0.49–1.62)	0.699	0.62	(0.19–2.00)	0.420	**1.71**	(1.01–2.87)	**0.044**
Dysphagia (odynophagia)	**0.45**	(0.22–0.92)	**0.029**	**2.34**	(1.08–5.05)	**0.030**	1.15	(0.42–3.11)	0.786	1.26	(0.31–5.15)	0.749	**2.46**	(1.05–5.76)	**0.038**
Rheum (runny nose)	0.68	(0.40–1.14)	0.144	0.76	(0.37–1.56)	0.454	1.13	(0.52–2.46)	0.754	NA	NA	NA	1.74	(0.93–3.25)	0.084
Cough	**0.60**	(0.38–0.97)	**0.037**	1.68	(0.95–2.97)	0.075	1.61	(0.66–3.92)	0.292	1.41	(0.53–3.79)	0.495	**2.20**	(1.18–4.12)	**0.014**
Hoarseness	**0.47**	(0.28–0.80)	**0.005**	1.49	(0.79–2.84)	0.219	1.10	(0.53–2.28)	0.802	0.25	(0.03–1.84)	0.174	1.64	(0.88–3.07)	0.123
Respiratory infection (A)	**0.56**	(0.41–0.76)	**<0.001**	1.23	(0.85–1.79)	0.270	1.18	(0.74–1.87)	0.483	0.58	(0.26–1.29)	0.180	**1.77**	(1.22–2.55)	**0.003**
Respiratory infection (B)	**0.54**	(0.33–0.88)	**0.014**	1.62	(0.91–2.87)	0.102	0.88	(0.46–1.67)	0.684	0.89	(0.28–2.89)	0.847	**2.79**	(1.49–5.22)	**0.001**
Ear pain	0.77	(0.49–1.21)	0.258	1.34	(0.79–2.26)	0.276	0.89	(0.47–1.65)	0.700	0.61	(0.18–2.00)	0.413	1.59	(0.91–2.76)	0.101
Ear infection (A)	0.68	(0.45–1.00)	0.052	1.32	(0.82–2.13)	0.261	0.85	(0.51–1.43)	0.547	0.85	(0.36–2.02)	0.717	**1.60**	(1.01–2.52)	**0.044**
Eye redness	0.71	(0.48–1.06)	0.096	1.55	(0.96–2.50)	0.072	1.31	(0.69–2.49)	0.407	1.32	(0.56–3.14)	0.526	0.94	(0.63–1.42)	0.782
Eye pain or burn	**0.55**	(0.34–0.88)	**0.013**	**2.05**	(1.19–3.51)	**0.009**	1.63	(0.77–3.48)	0.205	0.56	(0.17–1.86)	0.341	1.14	(0.66–1.95)	0.644
Tear secretion	0.62	(0.29–1.34)	0.223	0.98	(0.38–2.54)	0.973	1.10	(0.41–2.95)	0.847	0.30	(0.04–2.19)	0.233	0.77	(0.35–1.70)	0.522
Eye infection (A)	**0.63**	(0.45–0.89)	**0.008**	**1.52**	(1.01-2.30)	**0.047**	1.33	(0.79–2.25)	0.286	0.94	(0.42–2.13)	0.887	0.94	(0.65–1.36)	0.741
Eye infection (B)	0.80	(0.47–1.36)	0.415	**1.84**	(1.06–3.22)	**0.032**	1.68	(0.69–4.11)	0.254	0.62	(0.18–2.12)	0.446	0.86	(0.48–1.53)	0.605
Cutaneous infection	0.38	(0.12–1.25)	0.112	0.98	(0.20–4.80)	0.984	**0.31**	(0.11–0.88)	**0.027**	1.57	(0.35–6.94)	0.555	1.15	(0.39–3.37)	0.805
Medical consultation	0.92	(0.58–1.45)	0.712	1.33	(0.75–2.35)	0.328	**0.32**	(0.19–0.53)	**<0.001**	0.98	(0.33–2.92)	0.974	**2.51**	(1.40–4.50)	**0.002**
Medication receiving	0.80	(0.54–1.18)	0.259	1.00	(0.61–1.65)	0.993	0.76	(0.46–1.27)	0.301	0.81	(0.32–2.00)	0.641	**2.10**	(1.28–3.44)	**0.003**

OR, Odds Ratio; *p*, *p*-value; All above factors were included in the same logistic regression model adjusted for age, gender and residence, and clustering within households; ^a^ Swimming time: >60 min *vs*. ≤60 min; ^b^ Enterococci density: ≥35 cfu/100 mL *vs*. <35 cfu/Ml; ^c^ Bather density: B+C *vs*. A beach.

### 2.6. Need for Medical Consultation or Use of Medication

There were increased risks of receiving medical consultation (OR = 3.07, 95% CI 1.15–8.23) and of medication use (OR = 2.98, 95% CI 1.30–6.82) among bathers compared with non-bathers. However there were no significant differences in risks of hospitalization or home care in bathers compared with non-bathers (Table 3, Table 4).

## 3. Discussion

This study aimed to assess the incidences of symptoms related to GI, RI and ear eye and dermatological infections among bathers after exposure to recreational seawater that met the criteria for excellent bacteriological quality. All three beaches sampled conformed to the excellent water quality criteria for coastal waters set by the EU Directive 2006/7/EC and the water quality criteria for bacteria set by the EPA for recreational marine waters. We identified increased risks among bathers in recreational seawater compared with non-bathers for a series of symptoms related to RI GI ear and eye infections. 

Some evidence of increased rates of nausea/vomiting and abdominal pain were found among bathers compared with non-bathers in univariable analysis. A significantly increased risk of GI was found in bathers compared to non-bathers according to the more sensitive disease definition A as well as and some indication of increased risk was demonstrated using the more specific definition B. GI (gastroenteritis) constitutes the most frequent adverse health outcome associated with exposure to recreational water and increased risks for swimmers in relatively polluted marine waters compared with those for swimmers in relatively unpolluted water have been repeatedly reported in many epidemiological studies [16,17,18,19,20,21]. In the current study the risk of possible GI was estimated at between 3.16 (95% CI 0.95–10.52) and 3.60 (95% CI 1.28–10.03) depending on the disease definition (B or A). In other studies the risks of GI among bathers compared with non-bathers after exposure to excellent quality (fecal streptococcal densities = 0–39 cfu/100 mL) or relatively unpolluted waters (fecal coliform densities <300 cfu/100 mL) were 2.22 (not statistically significant) and 4.60 respectively [19,22]. In addition a combined relative risk of 1.36 (95% CI 0.91–2.03) of GI was estimated for marine water below the EPA standards [8]. A review article assessed the relative risk of GI after exposure to clean seawater as between 1.00–2.50 [7]. Moreover, in our study, higher rates of possible GI were observed among visiting bathers compared with local bathers, suggesting an influence of immune status on disease occurrence, as reported elsewhere [17]. However, increased rates of other bathing-related diseases have not been reported among visiting bathers in relation to local bathers (data not shown). Non-point, diffuse sources may be the contamination sources beyond any bather fouling. However, some discussion to the fecal contamination sources at the three beaches could be helpful regarding interpretation of our results. Beach A was a wide beach covered with sand, in front of a small village. The village did not maintain a main sewage system and untreated water-carried liquid waste were discharged in septic tanks at each home. Parallel to the beach and about 100 m behind the shore was an influent of a nearby river that flows into the beach. Beaches B and C had similar geophysical characteristics and were covered with loose stones (pebble). Taking all the above data into account and also and after a risk assessment procedure beach A was categorized as a high risk for water contamination in comparison to beaches B and C. Nevertheless, beach A was characterized by a lower bather density and lower incidence of symptoms in comparison to the beaches B and C.

Among the respiratory symptoms, only for sore throat there were some indications to be associated with bathing (OR = 4.28, 95% CI 1.35–13.51). An increased rate of RI among bathers compared with non-bathers was found using disease definition A (OR = 1.92, 95% CI 1.00–3.67). Other studies found the relative risks of respiratory symptoms after exposure to relatively unpolluted (fecal coliform densities <300 cfu/100 mL) or excellent quality waters (fecal streptococci density = 0–27 cfu/100 mL) of 2.40 and 1.65 (not statistically significant), respectively [3,23]. It is notable that Marion *et al*. observed elevated illness risks among swimmers in inland recreational waters where the water was relatively unpolluted with respect to fecal indicator densities [24].

As reported in other studies, the current study found that there was some evidence that bathers experienced increased ear problems compared to non-bathers, even after exposure to water with few fecal index organisms [23]. There was some indication that ear pain (otalgia) was associated with bathing (OR = 12.02, 95% CI 1.69–85.58) despite the uncertainty of this result due to the large range of confidence interval, and it is also the main symptom of ear infections such as acute otitis media or otitis external. Evidence of increased rate of ear infections among bathers compared with non-bathers was found using the more sensitive definition A (OR = 17.21, 95% CI 2.42–122.34). Other studies have reported relative risks of ear ailments after exposure to excellent quality water (fecal coliform density = 0–40 cfu/100 mL) and to relatively unpolluted waters (fecal coliform density <300 cfu/100 mL) of 2.36 and 4.30, respectively [3,22]. 

Eye redness and eye pain (or burning) was associated with bathing, and the incidence of eye infection was increased among bathers compared to non-bathers using both disease definitions (A and B). Another study found a relative risk of eye infection after exposure to relatively unpolluted water of 6.30 [22], compared to a risk of 2.43 and 3.48 for definition A and B respectively in our study.

Cutaneous infections are common after scratches or cutaneous cuts. However, our study, which was conducted in water with low levels of indicator bacteria, found no increased risk of cutaneous infections among bathers in relation to non-bathers. A recent analysis demonstrated that swimmers exposed to seawater with high levels of several indicator bacteria experienced significant increases in skin-related symptoms (irritation, rash, infection, itchiness, *etc*.) compared to non-swimmers [25]. A previous study reported cutaneous infections among bathers in recreational water, but the quality of water was not mentioned [26], while another study described cutaneous infections among bathers in water fulfilling the requirements of the 2006 EU Directive ([27], personal communication: Schets FM, date 28/12/2010). 

The significantly increased risks of medical consultation (visiting a doctor) or receiving medication (visiting a pharmacist or use of a medication) among bathers compared with non-bathers provides indirect proof of higher morbidity among bathers. However, there was no evidence for increased hospitalization or home care in relation to the above-described diseases, suggesting that the diseases or symptoms related to swimming in excellent quality seawater are mostly of mild or medium severity. An interesting finding of our study is the observed association between swimming time and reported symptoms of GI and respiratory infections, after controlling for several confounding factors. This finding would provide a basis for considering water as a causal pathway for symptom development among bathers. 

This study identified increased risks of possible infections among bathers, even though the bathers were swimming in excellent quality seawater. This finding is in line with other studies and suggests that factors other than water quality, such as the crowded conditions, bathing itself, or sand quality might be contributory factors. In particular, bather density on the beaches could explain many of the diseases observed, especially RIs [28]. Moreover, the measurement of bacterial indicators is a very coarse tool intended to evaluate the beach as a whole, and may be inadequate for indicating risk to a particular bather. Further studies are needed to provide more evidence for these factors. It should be mentioned that other studies and analyses by Ashbolt [29] and Fleisher [30] have supported a multiple indicator approach for the protection of public health among bathers. It is possible that the indicators that are bacteriological are useful for GI illness risk but not other ailments (eye, throat, respiratory). Viruses, such as adenovirus, may be important pathogens causing illness and may not measure or associate with bacteriological indicators. In addition, toxins produced by algae may also represent a contributor to elevated illness among swimmers. 

This study presents some limitations. Its questionnaire-based nature meant that it could have been subject to recall bias, as a result of the self-reporting of the symptoms by the participants. Moreover, the interviewers were not blinded, and the answers to the first part of the questionnaire were known to interviewers conducting the second part. In addition, bathers may be more likely to report illnesses than non-bathers. It is also possible that our definition of bathers, which included swimming for at least 10 minutes (body immersion) without head immersion could have led to an underestimation of risks (especially ear infection). The control group should ideally be representative of the exposed individuals in all respects, apart from the exposure. However, identifying such a control group in a beach environment in Greece is very difficult, because almost everybody who attends the beach also swims; the small proportion of people who attended the beach as non-bathers were often ill, very elderly, or disabled, or pregnant or menstruating women. Our method of selecting control individuals was therefore the best possible option. No questions regarding socioeconomic status were included in the questionnaire, and the control and bathing groups were not matched for age, gender or residence, though these factors were controlled for during the analysis. However, it remains a possible limitation of the study that some possible risk factors were not controlled for by either the study design or the analysis. Another possible limitation of the study was the small number of participants who were included in the control group (*n* = 572), which may have been the main reason for the low incidence of illnesses reported within the control group, affecting the statistical power and the fitting of multiple logistic regression models. Yet, the selected duration of follow up (10 days) was associated with both advantages and disadvantages; it reduced possible recall bias, but may also have resulted in an underestimation of infectious diseases with a longer incubation period (e.g., *Giardia lamblia* or *Cryptosporidium parvum*). However, waterborne parasites are very rare in Greece, especially in the study area [31]. In addition, the adoption of two or more criteria for each symptom area could increase the probability that one positive result will have arisen by chance. Further, the study population was mostly from the local area. It remains possible that the bathing group, if regular attendees at the beach, may have a degree of resistance to infections derived from prior exposure. The likely impact on the results would be an expected increase in symptoms among non-local persons. Last, the discrimination ability of the models does not appear to be satisfactory (AUC < 0.7). However, a low ROC value it is a subjective science, but does provide good information regarding the strength or limitations of our logistic regression models. 

## 4. Experiment Section

### 4.1. Study Beaches and Study Population

**Figure 1:**
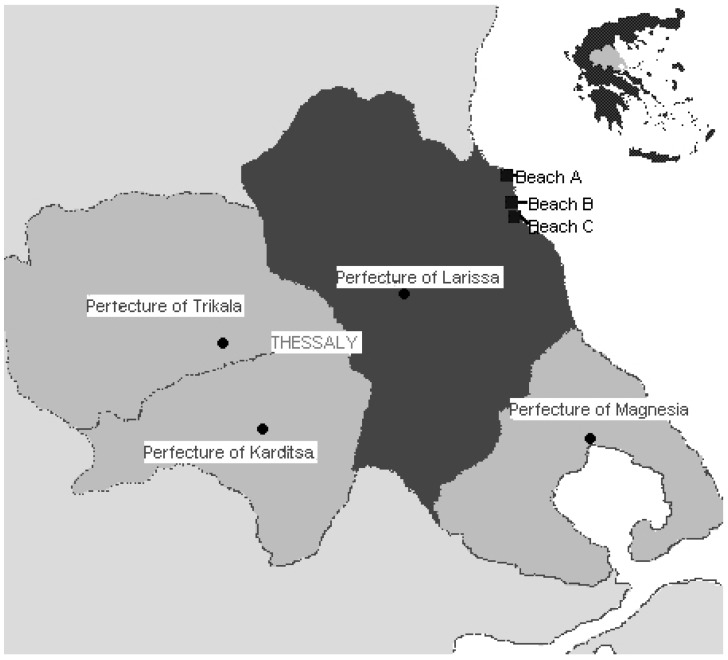
Marine beach sites.

A prospective cohort analytical epidemiological study was conducted during the summer bathing period in 2008, including bathers from three different bathing sites (beaches A, B, C) in the municipalities of Melivia and Evrymenes in the Prefecture of Larissa (Figure 1). According to data from the frequently monitored coastal bathing areas for a period of 10 years (1997–2006) and additional measurements carried out by the Department of Hygiene and Epidemiology of the University of Thessaly, all three beaches conformed to EU Guideline values, with water quality classified as excellent according to requirements for coastal water [10,11]. Beaches A and C were frequently monitored as part of the national surveillance program for water quality. The study was conducted only on weekends (Friday, Saturday and Sunday) when the beaches were presumed to be full of bathers, who could be recruited as volunteer participants.

For the purpose of this study, bathers were defined as people who swam for at least 10 min (body immersion), including those who had not immersed their head in the water. In order to limit the variation in composition of the bathers and to minimize biases, emigrants, foreign tourists, and members of the Roma community were excluded from the study. The exclusion of these groups was based on socioeconomic, cultural and behavioral differences that might influence the incidence of the diseases included in the study. However, only 10 members of the Roma community were found at the beaches. Consequently, we do not believe that their exclusion has biased the results of the present study. The enrolment of the bathers and non-bathers in the study lasted approximately 2 months.

A parallel control group of non-bathers was included in the study. Non-bathers were defined as people who had not visited a beach or swimming pool within the 15 days prior to the interview, and who lived in the same residence area as the bathers. This control group was selected randomly, mainly using the telephone directory for the local area and for the wider area of Thessaly, where the majority of bathers resident. A random digit dialing approach was adopted. Emigrants, Roma, and foreign tourists were also excluded from the control group. The study was approved by the Medical Department (Scientific) Committee of the University of Thessaly which is responsible for ethics (Helsinki/ Human Research Subject’s Committee).

### 4.2. Questionnaire and Data Collection

The questionnaire included 26 questions and was divided into two parts. The first part was completed during a face-to-face interview and included information on demographics (age, sex, residence, land line and cell phone numbers), bathing behavior (diving, bathing duration and time, playing in water or on the beach, use of sunscreen, umbrellas, food consumption, *etc*.), aesthetic appeal of the beach (garbage on the beach or at sea, presence of animals, *etc*.), and day of bathing. Individual questionnaires were completed for each eligible person in the household. In the case of families bathing together, all the members of the family were asked to participate. A follow-up interview was performed 10 days later to complete the second part of the questionnaire. The follow up was conducted by telephone interview, and included questions regarding symptoms of potential bathing-related diseases, such as respiratory infections (RIs), gastroenteritis, ear infections, eye infections and cutaneous (dermatological) infections. Participants were also asked if they had visited a doctor or a pharmacist, and if they have been hospitalized or received home care for any of the above described symptoms or diseases. The first interview was done at the beach before or after bathing.

Interviews were conducted by nine interviewers who were trained in techniques regarding approaching the bathers and completing the questionnaires. The interviewers comprised doctors, medical students and a nurse. The procedure was supervised by a senior medical doctor who provided additional help to the interviewers if necessary. 

The control group of non-bathers completed the questionnaire by telephone interview. The same questionnaire was used for bathers and non-bathers. Information regarding children was provided by their parents or other adults (e.g., grandparents).

### 4.3. Self-Reported Symptoms and Disease Definitions

To avoid errors due to disease definitions, bathers were asked to self-report symptoms, rather than diseases. The following symptoms were included in the questionnaire for each disease:

- Gastrointestinal infection (GI): nausea/vomiting, abdominal pain, diarrhea (defined as two or more loose or watery stools in a 24-hour period), fever.
- RI: sore throat, dysphagia (odynophagia, pain during deglutition), rheum, cough, hoarseness. Respiratory illness in our study included infection of the upper or lower respiratory organs.
- Ear infection: ear pain, sense of fullness in the ear, otorrhea, ear itching.
- Eye infection: Red eye (redness of the conjunctiva), eye pain/burning, eye secretion, mucopurulent exudate.
- Symptoms possibly related to cutaneous infection (e.g., self-reported symptoms as rash).

To improve the accuracy of the analytical results, two definitions were used for each disease (RI, GI, ear and eye infection); one sensitive (definition A) and one more specific (definition B). Definition A required a positive answer to one symptom in a disease category, while definition B required positive answers to two or more symptoms in a disease category. Fever as a single symptom was not included in definition A for GI, but could be included in definition B for GI.

### 4.4. Microbiological Water Quality Assessment

Water samples for quantitative analysis of bacterial indicators were collected from the three different bathing sites on the same day that the interviews took place. Beaches A and B each had three sampling points while beach C had only one, because of its small size. One sample was collected from each sampling point (seven sampling points in total) in the morning between 10:30 a.m. and 12:30 p.m. A standardized form was used to record: bather density, water temperature (in degrees Celsius), presence of high waves, wind direction, phenolic smell, garbage, wrack, and oil or tar on the beach or sea. The water samples were quantitatively analysed for microbiological parameters the same day. Water samples were tested for intestinal enterococci, *E. coli*, fecal coliforms, total coliforms and *Staphylococcus aureus*. The water sampling methodology has been described in detail in a previous publication [28]. 

### 4.5. Statistical Analysis

The relationships between bathing exposure and symptoms were analysed. Our data were derived from survey sample data where the participants were not independently sampled, since members of the same household (clustering effect of household) could respond as well, affecting, consequently, the results. To account for the clustering effect in the univariable and multivariable analysis we included the variable “cluster” where participants of the same family belonged to the same cluster while every individual participant belonged to a different cluster, and then we conducted Chi-square test or Logistic Regression through Complex Samples of SPSS.

In univariable analysis, Pearson Chi-square test or Likelihood Ratio test were used and in multivariable analysis, multiple logistic regression analysis for survey sample data were conducted. Symptoms or diseases which were deemed by univariable analysis to be statistically significant in their association with bathing with bathing were included as dependent variables (outcome variables) in multivariable logistic regression analysis controlling for the confounders age, sex, residence, duration of exposure to water, food consumption, head immersion and levels of Enterococci.

Outcome variables were coded in models as 1 (presence of symptom or disease) or 0 (absence of symptom or disease). Age was inserted as a continuous variable, while gender and residence were used as dummy variables (male = 1, female = 0; visitors = 1, locals = 0). Relative risks (RR) and adjusted odds ratios (OR), and their corresponding confidence intervals (CI) were calculated. A *p*-value less than 0.05 was considered statistically significant [32,33,34].

In univariable analysis, in cases where non-bathers had a frequency of zero in a cell we applied the Haldane correction in order to allow the calculations of the relative risks among bathers in relation to non-bathers. The Haldane correction (or Haldane method) is used to avoid errors in the calculations some of the Chi-square test by adding 0.5 (1/2) to each cell of a contingency table [32,35]. However, variables with zero frequency were not included in the logistic regression models. ROC analysis was conducted to assess the discrimination ability of each model by measuring the area under the ROC curve (AUC).

Statistical analysis was performed using EPI-INFO software, version 3.4.3 (Centre for Disease Control and Prevention, Atlanta, GA, USA), SPSS version 16.0 (SPSS Inc., Chicago, IL, USA) and STATA version 10.0 (StatCorp LP, College Station, TX, USA).

## 5. Conclusions

This study provides evidence of increased risks among bathers in recreational seawater of symptoms related to GI, RI, and ear and eye infections. Symptoms were more common in people who swum for longer than in those who swum for a shorter period. These results were observed despite the fact that the water on the sampled beaches met the bacterial water quality criteria set by EU directives and the EPA. It seems that much remains to be learned about predicting illness among bathers, as in the setting of the beaches studied, no associations between microbes and health outcomes were apparent. These results suggest that risk-assessment in recreational marine waters could be a more inclusive approach for the protection of public health in comparison to numerical compliance to bacteriological indicators alone. Ways of identifying beaches where enterococci is not predictive, and finding alternative health predictors could be important next steps.
